# Dermoscopy as an adjuvant tool for detecting skin leiomyomas in patient with uterine fibroids and cerebral cavernomas

**DOI:** 10.1186/1471-5945-14-7

**Published:** 2014-04-16

**Authors:** Laura Diluvio, Claudia Torti, Alessandro Terrinoni, Eleonora Candi, Raffaella Piancatelli, Emilio Piccione, Evelin Jasmine Paternò, Sergio Chimenti, Augusto Orlandi, Elena Campione, Luca Bianchi

**Affiliations:** 1 Department of Dermatology, University of Tor Vergata, Rome, Italy; 2 Department of Experimental Medicine, University of Tor Vergata, Rome, Italy; 3 Department of Gynecology and Obstetrics, University of Tor Vergata, Rome, Italy; 4 Dermatologist in Rome, Rome, Italy; 5 Department of Anatomic Pathology, University of Tor Vergata, Rome, Italy

**Keywords:** Cutaneous leiomyoma, Dermoscopic pattern, Dermatofibroma, Differential diagnosis

## Abstract

**Background:**

Hereditary syndromes frequently need the cooperation of different specialties to increase diagnostic competence. Multiple cutaneous and uterine leiomyomatosis syndrome is a rare autosomal dominant disorder caused by the mutations of the fumarate hydratase gene, demonstrated in 80 to 100 percent of affected individuals. This can be linked to an increased risk of renal cancer in both sexes. The skin involvement is described to highlight the diagnostic role of the cutaneous counterpart in identifying this rare syndrome.

**Case presentation:**

A 37-year-old woman suffering from several uterine fibroids presented multiple, painful, papulo-nodules on her left subscapular side, both forearms and legs. The patient underwent surgery on six lesions: five were leiomyomas, whilst one was a dermatofibroma. Genetic sequencing did not evidence known fumarate hydratase gene mutations. Dermoscopy showed a brown delicate pigmented network and included leiomyomas among the non-melanocytic benign skin tumours featuring a dermatofibroma-like pattern. Abdominal computerized-tomography scan did not reveal renal cancer, but brain magnetic resonance imaging showed one asymptomatic cerebral cavernoma. The patient benefited from the surgical removal of the five larger cutaneous lesions and from gabapentin, which relieved her pain.

**Conclusions:**

This observation highlights the usefulness of dermoscopy in the diagnosis of cutaneous leiomyomas disclosing multiple cutaneous and uterine leiomyomatosis syndrome. Dermoscopy should be performed for non-melanocytic multiple lesions mimicking leiomyomas in a large number of patients, to establish a strict classification and identify false negative cases or evaluate them as dermatofibromas. In this case, the dermatologist recognized the risk of renal cancer and cerebral cavernomas.

## Background

Multiple cutaneous and uterine leiomyomatosis (MCUL), previously known as Reed's syndrome, is an autosomal dominant disease with incomplete penetrance characterized by the onset of uterine leiomyomas or fibroids and skin leiomyomas in both sexes [1,2]. The coexistence of cutaneous and uterine leiomyomas may cluster papillary type2 renal-cell carcinoma or renal collecting duct cancer, codifying a recent variant appointed as hereditary leiomyomatosis and renal cell cancer (HLRCC) [3]. Even though different gene mutations, missense, nonsense or whole gene deletions have been described in MCUL syndrome, a definite association between site or type of mutation and the risk of papillary renal-cell carcinoma has not been found yet [4]. Cutaneous leiomyomas - accounting for 75% of all extra-uterine leiomyomas - are uncommon benign smooth muscle tumours deriving either from the erector pili muscle of the pilosebaceous unit (piloleiomyomas), the cutaneous vascular smooth muscle fibers (angioleiomyomas) or from the dartos muscle (genital leiomyomas) [5]. Piloleiomyomas are the most common form and show firm, skin-colored or pink-brown soft dermal papules or nodules, ranging up to 2.0 cm in diameter, distributed in single, clustered, segmental or disseminated patterns. Linear or segmental leiomyomas may present a dermatomal-like distribution [6]. Common locations include trunk, extensor surface of the extremities and face, more often in adulthood then in childhood. Neoplasms can be asymptomatic or quite debilitating and actually painful mainly in response to pressure or cold temperature [7]. Clinical differential diagnoses include several skin lesions, such as dermatofibroma, eccrine spiradenoma, neurofibroma, angiolipoma, neurilemmoma, glomus tumour, keloid, hamartomas and blue rubber bleb nevus syndrome when painful, or dermal nevus, trichoepithelioma, lipoma, cylindroma or poroma among the asymptomatic tumours [8]. The skin involvement of a case of MCUL syndrome is reported to support the diagnostic role of the cutaneous counterpart in identifying this rare syndrome. We present and discuss a case of multiple cutaneous lesions and the usefulness of dermoscopy for a better understanding of skin leiomyomas in the wide spectrum of non-melanocitic lesions.

## Case presentation

**Figure 1:**
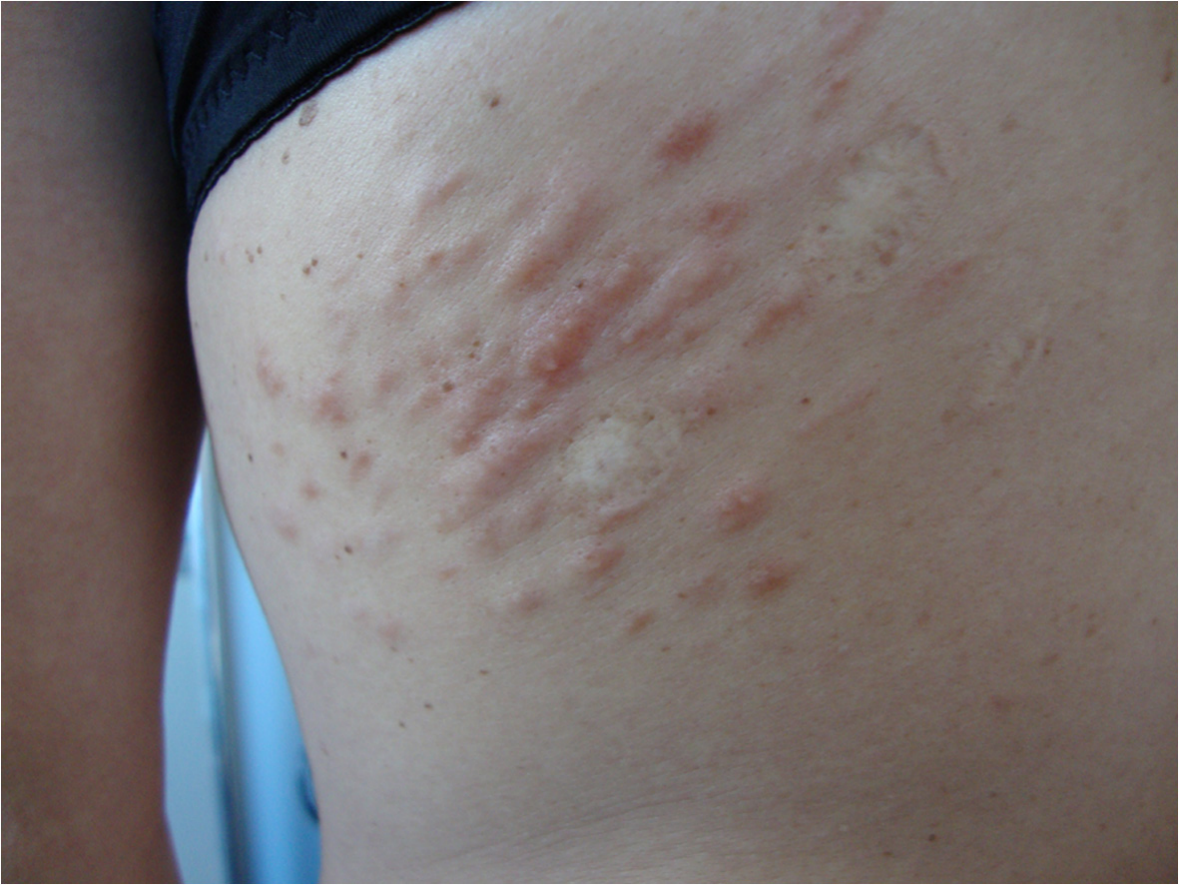
**Clinical picture of the patient.** Pink-to-brown papules and nodules on her left sub scapular region and both forearms.

**Figure 2:**
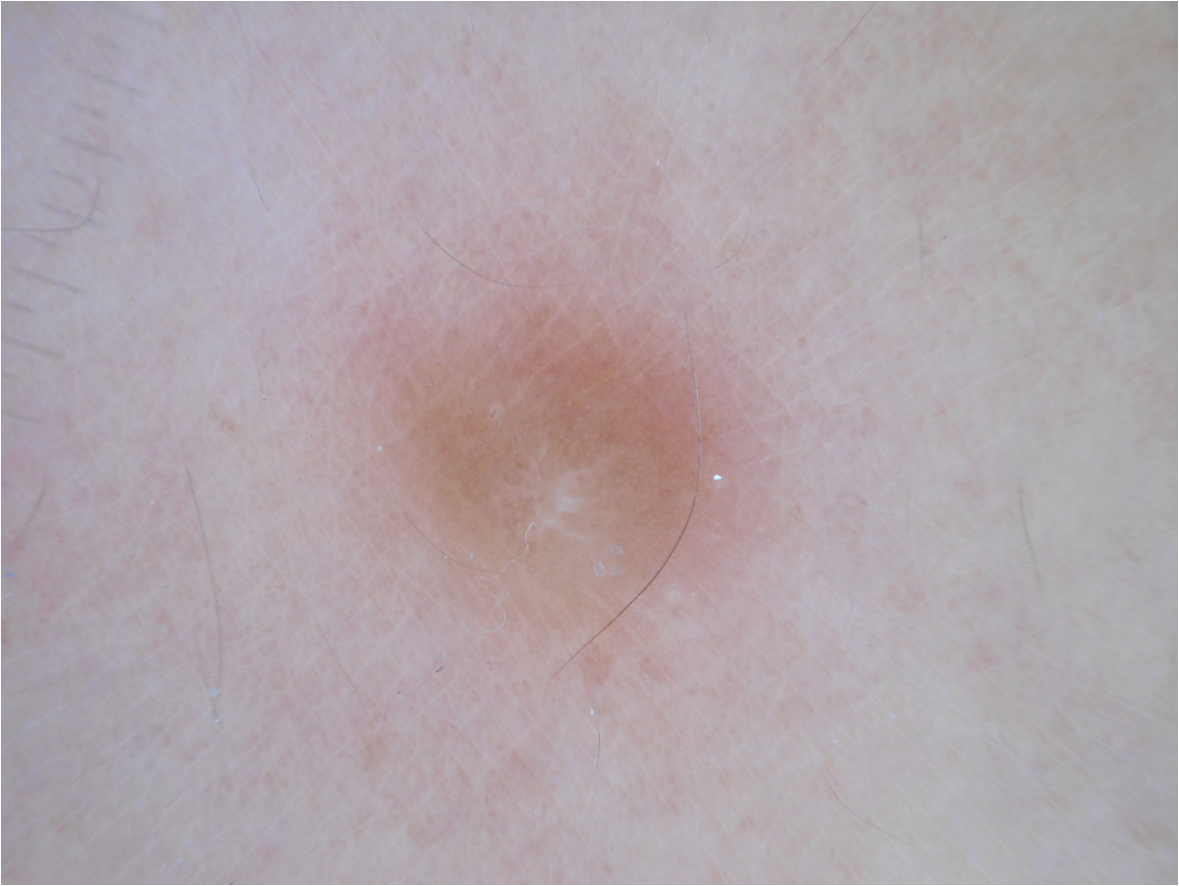
**Dermoscopic feature of dermatofibroma of the patient.** Central white scar like patch with delicate pigment network in dermatofibroma of the leg.

**Figure 3:**
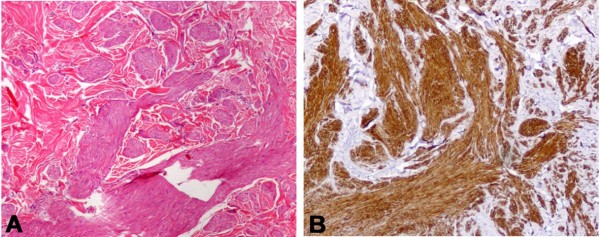
**Morphological and immunohistochemical myocitic aspect of a skin leiomyoma. (A)** Hematoxilyn-Eosin stained section reveals a poorly circumscripted lesion from interlacing bundles of benign smooth muscle cells; **(B)** Immunohistochemistry confirms the positivity for α-smooth muscle actin of leiomyoma cells. Original magnification, ×200; B, Diaminobenzidine as chromogen.

A 37-year-old woman complained of an 18-year history of progressive onset of multiple, firm, smooth, soft, pink-to-brown papules and nodules on her left sub scapular region and both forearms, ranging from 1 to 2 cm in diameters. Some lesions were arranged in dermatomal-like clustered nodules and plaques on her left upper back (Figure 1). The lesions could become symptomatic after mechanical injury, pressure, cold thermal stimuli or emotional stress. Moreover, we observed brownish nodular lesions on her leg similar to a dermatofibroma, displaying the most common pattern (Figure 2). Past medical history included early menarche, dismenorreas, menometrorrhagia, anaemia and uterine fibromatosis with myomectomy in 2000. Her family history did not reveal similar skin lesions in kindred nor any history of uterine diseases, hysterectomies or renal cancers. A total of 50 cutaneous lesions were counted in our patient; the patient had already removed a large cutaneous leiomyoma in 2003. Five larger nodules were surgically removed to solve the pain and distress caused by their size. Histology confirmed the diagnosis of cutaneous piloleiomyomas. Our main finding was a dermal proliferation of irregular interlacing bundles of benign smooth muscle cells with minimal atypia and scarce proliferative activity (Figure 3A). Antibodies against α-smooth muscle actin (Figure 3B), desmin and myosin positively stained the neoplastic growth. Moreover, the histological examination of the leg lesion confirmed the presence of dermatofibroma.

Genetic analysis was performed using RNA extracted from a leiomyoma biopsy. Complete coding region sequencing of all 10 exons of *Fumarate Hydratase* gene (Gb #NM_000143) was evaluated using direct sequencing of RT-PCR amplified FH transcript. The sequence did not reveal nucleotide changes in coding region, due to the presence of amino acid substitutions. This does not exclude the possible presence of complex genomic mutations located inside introns, which could lead to abnormal transcripts. The level of enzymatic FH analysis should be investigated to further characterise the patients for FH activity depletion. Complete routine blood testing and urinalysis did not show anomalies. Abdominal computerized-tomography (CT) scan displayed uterine fibroids and normal appearing kidneys. Brain magnetic resonance imaging (MRI) showed one asymptomatic cerebral cavernoma (data not shown).

**Figure 4:**
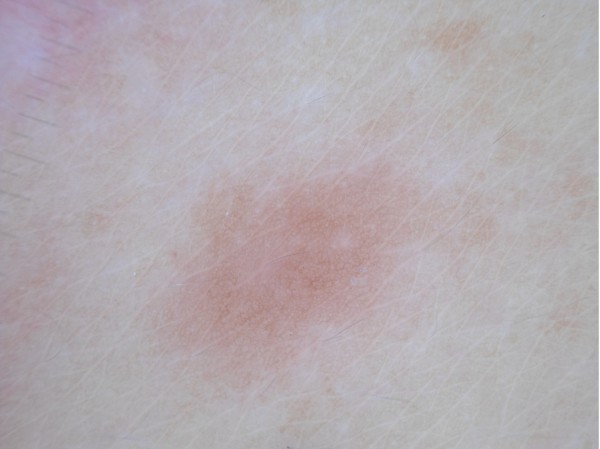
**Dermoscopic feature of leiomyoma.** Delicate dermatofibroma-like pigment network with thin lines of pinkish-light brown colour and regular meshes in all lesions.

**Figure 5:**
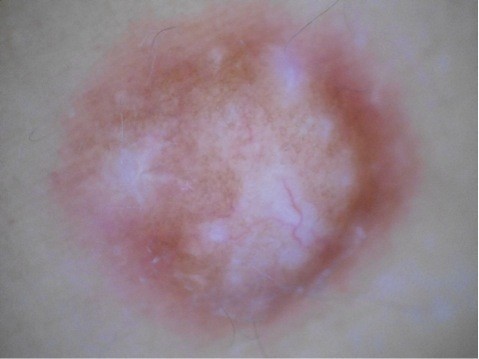
**Different dermoscopic feature of leiomyoma.** White cloud-like area without scar features.

Dermoscopy was performed using Dermlite ® with non–contact polarized light on each cutaneous leiomyoma. A delicate dermatofibroma-like pigment network - due to thin lines of pinkish-light brown colour and regular meshes - was present in all lesions (Figure 4). The network was regularly distributed and gradually faded into the surrounding skin. No vascular structures were observed. In some lesions, a white cloud-like area without scar features was observed (Figure 5). As expected, reflectance confocal microscopy (RCM) did not show any remarkable findings due to the deep dermal location of the proliferation. Leiomyoma-related discomfort significantly improved with gabapentin - started from 75 mg/die and then increased up to 225 mg/die for 1 month - with marked reduction of the pain.

## Conclusions

Hereditary syndromes frequently need the cooperation of different specialties, and MCUL/HLRCC disorder is a relevant example of this occurrence. The skin is often part of hereditary syndromes. Alongside the clinical and histological approach, we investigated if, in vivo, non-invasive, methods of investigations, dermoscopy or RCM, could increase our diagnostic skills. Cutaneous leiomyomas are dermal proliferations of benign smooth muscle fibres; in these cases, the diagnostic role of superficial investigations is regarded as possibly uncertain. A new interesting finding came from dermoscopy, which showed also leiomyomas among the non-melanocytic benign skin tumours featuring a dermatofibroma-like appearance. In all leiomyomas we observed a delicate pigment network with thin lines of pinkish-light brown colour and regular meshes, regularly distributed and gradually faded into the surrounding skin. The larger lesions could present vascular structures, although they were not representative for most piloleiomyomas (Figure 4). In some piloleiomyomas we documented masses of “white like-cloud” areas without translucent streaks, typical of the dermoscopic central scar-like patch in dermatofibroma (Figure 5). However, as also recently described by Paschoal, we did not see hyperpigmented circular and/or elongated structures within the central hypopigmented area [9].

The pigmented network pattern seems reasonably related to a reactive epidermal basal hyperpigmentation, as observed in the histologically confirmed lesions, due to the lower neoplastic growth of dermatofibroma and leiomyoma. RCM, as expected, did not give consistent results because of the deep dermal location of the proliferation.

Surprisingly, our patient presented also a dermatofibroma, characterized by a white scar like patch surrounded by a delicate pigment network (Figure 2); this gave us the opportunity to better distinguish the dermoscopic findings and to establish specific criteria for differential diagnosis [10]. Before a pigmented network pattern of clinically non-melanocytic lesions, the dermoscopic differential diagnosis should consider seborrheic keratosis, solar lentigo, dermatofibroma, and supernumerary accessory nipple [11].

Although screening guidelines for MCUL/HLRCC syndrome have not been defined yet, Smit listed practical criteria to ensure an appropriate diagnostic approach [12]. Suspected MCUL should always motivate a histological diagnosis of at least one cutaneous lesion. DNA analysis to test FH mutations should be recommended for all patients with familiar or severe cutaneous or uterine leiomyomatosis to eventually detect an occult renal malignancy, with genetic counselling in positive cases. Furthermore, since some mutations could not be detected via standard sequencing analysis, an accurate enzymatic test should be developed to verify FH activity in patient cells. An accurate follow-up should include annual cutaneous and gynaecological examinations for the risk of leiomyosarcoma, together with annual renal ultrasound investigations. Furthermore, since cerebral angiomatosis has been also described in MCUL patients, dermatologists and gynaecologists should consider the relative risk of cerebral haemorrhages through brain MRI [13]. If in females coincident uterine fibroids with often multiple and segmental cutaneous leiomyomas suggest the diagnosis of MCUL syndrome, in males, the most common solitary appearance of the cutaneous lesions and the obvious absence of the gynaecological counterpart, make the diagnostic suspicion difficult, thus amplify the risk of a late diagnosis of a possible renal involvement. Besides surgery for large or painful lesions, nifedipine, phenoxybenzamine, nitroglycerine, doxazosin, gabapentin, topical lidocaine or capsaicin, carbon dioxide laser ablation or botulinum toxin injections, have been variously tested to improve the symptoms [14]–[16].

Our observation highlights the role of dermoscopy in the diagnosis of cutaneous leiomyomas disclosing this hereditary syndrome for the risk of renal cancer and cerebral cavernomas. Dermoscopy should be performed on non-melanocytic multiple lesions mimicking leiomyomas in a large number of patients, to establish a strict classification, in order to identify false negative cases or evaluate them as dermatofibromas. The role of the dermatologist is crucial in the final diagnosis of this rare syndrome in a patient with multi organ involvement. Therefore, dermoscopy can be considered as an adjuvant tool that gives some extra information before histology.

## Consent

Written informed consent was obtained from the patient for publication of this case report and any accompanying images. A copy of the written consent is available for review by the Editor of this journal.

## Competing interests

The authors declare that they have no competing interests.

## Authors’ contributions

LD has made substantial contributions to conception and design. CT has been involved in drafting the manuscript. AT carried out the molecular genetic studies. EC carried out the molecular genetic studies. RP made substantial contributions to acquisition of data. EP made substantial contributions to conception and design. EJP made substantial contributions to acquisition of data. SC made substantial contributions to conception and design. AO has made substantial contributions to revising it critically for important intellectual content. EC has made substantial contributions to revising it critically for important intellectual content. LB has given final approval of the version to be published. All authors read and approved the final manuscript.

## Pre-publication history

The pre-publication history for this paper can be accessed here:

http://www.biomedcentral.com/1471-5945/14/7/prepub
